# Chronic kidney disease screening and associated factors among people living with HIV on anti-retroviral therapy in Dar Es Salaam, Tanzania: Hospital-based cross-sectional study

**DOI:** 10.1371/journal.pone.0352024

**Published:** 2026-06-22

**Authors:** Magreth Theophil, Ally Abdul Lyimo, Joel Seme Ambikile

**Affiliations:** 1 Department of Internal Medicine, Nephrology Unit, Mwananyamala Regional Referral Hospital, Dar es Salaam, Tanzania; 2 Department of Clinical Nursing, Muhimbili University of Health and Allied Sciences, Dar es Salaam, Tanzania; 3 Department of Midwifery, Child and Reproductive Health, Muhimbili University of Health and Allied Sciences, Dar es Salaam, Tanzania; University of Cape Town Faculty of Science, SOUTH AFRICA

## Abstract

**Background:**

Chronic Kidney Disease (CKD) is a significant global health challenge, particularly among people living with HIV (PLHIV) on antiretroviral therapy (ART). The combined effects of HIV infection, long-term ART exposure, and other comorbidities increase CKD risk in this population. Despite international and national recommendations for routine CKD screening, the uptake remains inconsistent in resource-limited settings like Tanzania. Understanding the prevalence and associated factors of CKD screening is vital for improving early detection and patient outcomes. This study aimed to determine the prevalence of CKD screening and associated factors among PLHIV on ART in Dar es Salaam, Tanzania.

**Methods:**

This was a hospital-based cross-sectional study conducted in Dar es Salaam, Tanzania. Data were collected using a semi-structured questionnaire and the review of medical records. A systematic random sampling method was used to recruit participants in the study. Descriptive analysis was used to summarize participants’ characteristics and the prevalence of CKD screening. Univariate and multivariate logistic regression were applied to identify factors associated with CKD screening, with a p-value < 0.05 considered for statistical significance.

**Results:**

Among 426 adults on ART, CKD screening uptake was high (94.1%). Screening was more likely among participants with higher CD4 counts (>350 cells/µL; AOR: 9.73, 95% CI: 1.21, 78.16; p = 0.032), and less frequent ART clinic attendance was associated with lower screening odds (every six months vs. monthly; AOR: 0.16, 95% CI: 0.05, 0.48; p = 0.001). Interestingly, participants reporting provider recommendations for CKD screening had lower odds of screening (AOR: 0.17, 95% CI: 0.04, 0.79; p = 0.024). Sociodemographic factors, viral load status, comorbidity, perceived adequacy of CKD information, and distance to screening facilities were not significantly associated with CKD screening.

**Conclusion:**

CKD screening uptake among adults on ART was high, but screening was influenced by clinical and service factors. Higher CD4 counts and more frequent clinic attendance were associated with increased screening, while provider recommendations were paradoxically linked to lower screening, highlighting gaps in translating advice into action. Strengthening integrated, patient-centered approaches and ensuring systematic CKD screening across all patient groups is essential to optimize early detection and management of kidney disease in PLHIV.

## 1. Introduction

Chronic Kidney Disease (CKD) is a progressive condition affecting approximately 850 million people worldwide [1]. It contributes significantly to morbidity and mortality, particularly among people living with HIV (PLHIV) on antiretroviral therapy (ART), who are at increased risk due to HIV related and treatment-related factors [2,3]. Tanzania carries a substantial burden of HIV, with an estimated prevalence of 4.6% among adults aged 15–49 years, and of these, 62% are aware of their status, and 90% of those are on ART [4].

Worldwide, approximately 39.9 million individuals are living with HIV, with a significant proportion facing an increased risk of developing CKD due to HIV-associated nephropathy, ART nephrotoxicity (particularly Tenofovir), and co-existing conditions such as hypertension and diabetes [5–7]. The prevalence of CKD among PLHIV varies widely across sub-Saharan Africa with a pooled estimates of 12%. In East African countries, reported prevalence ranges from approximately 8% to 22%, with most studies estimating rates between 10% and 18% in Kenya, Uganda, and Ethiopia [8]. In Tanzania, CKD prevalence in the general population ranges between 7% and 14%, with higher rates observed in people living with HIV (PLHIV) [9–11].

Screening for CKD among PLHIV on ART using recommended methods, such as serum creatinine-based estimation of glomerular filtration rate (eGFR) and the urine albumin-to-creatinine ratio (UACR) for detection of proteinuria or albuminuria, is crucial for early diagnosis and management, as untreated CKD can result in substantial morbidity and mortality [12,13]. International and national guidelines recommend routine screening for CKD at the initiation of ART, with periodic annual follow-up thereafter, due to the increased risk of kidney impairment associated with HIV infection and prolonged exposure to ART [2,14,15]. Early identification enables timely interventions and referral to nephrology services, thereby improving clinical outcomes and reducing healthcare costs [16].

Despite the availability of guidelines emphasizing the importance of CKD screening for PLHIV on ART, the uptake remains suboptimal in many resource-limited settings [17]. Several studies mentioned limited diagnostic tools, inconsistent follow-up mechanisms, workforce shortages, and insufficient public awareness as the causes of poor adherence to CKD screening among PLHIV [18,19]. The government of Tanzania has strengthened the CKD screening through integration into the National Non-Communicable Diseases (NCD) Strategic Plan, capacity building of healthcare providers, decentralization of services, and incorporation of CKD screening into HIV care services [11]. However, persistent resource constraints and knowledge gaps among healthcare providers continue to pose significant challenges to effective CKD screening and management [19]. Without effectively addressing these challenges, the burden of CKD-related complications among PLHIV is likely to increase, resulting in higher morbidity and mortality and placing additional strain on the healthcare system [18,19]. Generating evidence on the availability and trends of CKD screening is therefore essential to inform the formulation and implementation of effective screening policies. In Tanzania, there is a paucity of studies focusing on CKD screening among PLHIV. Accordingly, this study aimed to determine the prevalence of CKD screening and identify factors influencing its uptake, to improve early detection, patient outcomes, and informed allocation of healthcare resources.

## 2. Materials and methods

### Study design

The study utilized a quantitative hospital-based cross-sectional study design to determine the prevalence of CKD screening and associated factors among people living with HIV (PLHIV) on antiretroviral therapy (ART).

### Study area

The study was conducted in Dar es Salaam, Tanzania’s largest commercial and administrative city, located along the eastern coast of the country on the Indian Ocean. The city serves as the country’s main economic hub and an important center for trade, transportation, and health services. Dar es Salaam has a well-established healthcare system, comprising tertiary hospitals, regional and district hospitals, health centers, and dispensaries that provide HIV care and treatment services. Tanzania has an estimated 1.6 million PLHIV, with Dar es Salaam accounting for approximately 18% of this population, making it an appropriate setting for this study [20].According to the National AIDS Control Programme (NACP), 179,596 PLHIV are currently receiving ART in Dar es Salaam, of whom 72% are female and 28% are male (National AIDS Control Programme, 2024). The study was conducted at three Regional Referral Hospitals within Dar es Salaam: Mwananyamala Regional Referral Hospital (MRRH), Temeke Regional Referral Hospital (TRRH), and Amana Regional Referral Hospital (ARRH). According to the District Health Information System 2 (DHIS2) 2025, annually, MRRH, TRRH, and ARRH provide services to 6630, 6352 and 6376 PLHIV, respectively [21].

### Study population and inclusion criteria

The study population comprised PLHIV on ART, aged 18 years and above, who had been receiving ART for at least 1 year and attended the HIV Care and Treatment Clinic (CTC) in the selected facilities. Participants were required to have clinical and laboratory records documenting CKD screening and to provide written informed consent to participate in the study. Individuals who were critically ill, mentally unfit, and or had incomplete medical records, specifically those with missing information on CKD screening status, were excluded from the study.

### Sample size and sampling technique

The study sample size was estimated to be 426 participants, calculated using the Cochran formula. Due to scarcity of data on the prevalence of CKD screening among PLHIV, a default prevalence of 50% was used. The calculation assumed a 95% confidence interval (Z = 1.96) and included a 10% adjustment for potential non-response.

The study was conducted in all three regional referral hospitals in the Dar es Salaam region, which were purposively selected due to their role in providing specialized HIV care and diagnostic services, including laboratory testing and nephrology consultations.

Participants were selected using a systematic random sampling technique. Daily clinic attendance lists and appointment registers were used as sampling frames. The total sample size was equally distributed across the three hospitals (142 participants per hospital). With an average of about 600 eligible patients per hospital during the data collection period, a sampling interval of 4 (***k*** = 4) was calculated using a formula “𝑘**=**𝑁**/**𝑛**”,** where N is the total number of eligible patients attending the CTC during the study period, and “n” is the required sample size per hospital. A random starting point between 1 and 4 was selected, and every fourth patient was recruited until the required sample size was achieved in each hospital.

### Data collection

#### 2.5.1. Data collection tools.

Data were collected using a structured questionnaire adapted from similar previous studies [22,23]. Some changes were made to the original tool to better serve the objectives of this study, through a systematic process that included a literature review of existing CKD screening instruments and framework-guided item modification. Content validity was ensured by assessing the relevance of each item, and construct and context validity were also considered to examine whether the items reflected the CKD screening practices within the HIV care settings in Tanzania. The validation process was conducted through expert review by a panel of three specialists in nephrology and HIV care, who independently reviewed the tool and provided feedback on relevance, clarity, and completeness. The modified questionnaire was piloted with 30 participants, and refinements made based on feedback. Some of the modifications involved the comorbid diseases assessed, the participants’ perception of CKD screening, and health system factors such as health care providers’ approach to screening. The tool was translated from English into Kiswahili to facilitate administration. It consisted of 28 items across three main domains, with an overall reliability coefficient of 0.88. The first domain captured socio-demographic data, including age, sex, education level, marital status, and occupation. The second domain included 6 items collecting clinical information, including duration on ART and presence of comorbidities. The third domain contained 8 items focused on health system factors, including the availability of screening services, healthcare provider recommendations, and perceived barriers to screening. Additionally, medical records were reviewed to obtain each participant’s CKD screening history within the preceding 12 months, including any prior kidney disease diagnoses made during the same period.

#### 2.5.2. Study variables.

In this study, CKD Screening was the dependent variable, defined as documentation of at least one diagnostic procedure used for CKD evaluation since initiation of ART, including serum creatinine test for estimating eGFR, urinalysis for detection of proteinuria or albuminuria, and/or renal ultrasound, where applicable/clinically indicated. The prevalence of CKD screening was determined as the proportion of PLHIV on ART who had undergone at least one of the CKD screening in the past year, in accordance with National HIV Management Guidelines (NACP) and the adult HIV care and treatment guidelines, which align with WHO recommendations [24]. Data on CKD screening were extracted from medical records to ensure accuracy. Participants were categorized as screened if there was documented evidence of at least one CKD screening procedure during the specified period, and as not screened if no such documentation was found within this period.

Independent variables in this study were potentially associated with CKD screening, including age, duration on ART, presence of comorbidities (e.g., diabetes, hypertension), and health system-related factors.

#### 2.5.3. Data collection procedure.

Data were collected from April to May 2025 with the assistance of two Research Assistants (RAs) who had health professional backgrounds. The RAs underwent a two-day training on the study objectives, data collection procedures, and ethical considerations. Permission to collect data at the selected hospitals was obtained from the Medical Officers in charge (MOI) prior to engaging the In-charges of the CTC.

Eligible participants were informed about the study objectives, procedures, and confidentiality assurances, and participation was entirely voluntary. Consenting participants were interviewed at the clinic after completing their routine consultation appointments. Interviews were conducted in a separate, quiet room to ensure privacy, and no personal identifiers were recorded. Completion of the questionnaire for each participant took approximately 20–30 minutes.

### Data management and analysis

Completed questionnaires were checked daily for completeness and consistency. Data were analyzed using the Statistical Package for the Social Sciences (SPSS) version 25. Descriptive statistics, including frequencies, percentages, means, and standard deviations, were used to summarize participants’ characteristics and the prevalence of CKD screening. Bivariate logistic regression analyses were performed to assess associations between each independent variable and the outcome variable (CKD screening status). Variables with a *p*-value ≤0.2 in the bivariate analysis were entered into a multivariable logistic regression model to identify independent predictors of CKD screening and to control for potential confounders. Statistical significance was determined at a 95% confidence interval with a p-value of <0.05.

### Ethical considerations

Ethical approval for this study was obtained from the Muhimbili University of Health and Allied Sciences (MUHAS) Institutional Review Board (IRB) (Ref. MUHAS-REC-02-2025-2667). Permission to conduct the study was granted by the respective health facilities. Written informed consent was obtained from all participants prior to data collection. Participant confidentiality was ensured through anonymization of data and secure storage of study records. Participants were informed of their right to withdraw from the study at any time without any effect on the care they received.

## 3. Results

### Socio-demographic characteristics of the study participants

A total of 426 participants were enrolled in the study. The mean age of participants was 42.5 years (SD = 11.5), with more than half (61.0%) aged 40 years and above. Females constituted 66.7% of the study population. More than half of participants (58.0%) had a partner (married or cohabiting), and almost half (49.5%) had attained secondary-level or higher education. Approximately four-fifths (81.5%) of participants were employed or self-employed, and the majority (87.1%) resided in urban areas (Table 1).

**Table 1:** Socio-demographic characteristics of the study participants (N = 426).

Variable	Frequency (n)	Percent (%)
Age		
Less than 40	166	39.0
40 and above	260	61.0
Sex		
Female	284	66.7
Male	142	33.3
Marital status		
No partner	179	42.0
Has partner	247	58.0
Level of education		
Primary or lower	215	50.5
Secondary or higher	211	49.5
Employment		
Unemployed	79	18.5
Employed/self-employed	347	81.5
Residency		
Peri-Urban	55	12.9
Urban	371	87.1

### Prevalence of CKD screening and clinical characteristics of participants (N=426)

Table 2 summarizes participants’ clinical characteristics and health information. The majority (n = 402, 94.1%) of participants had been screened for CKD in the past 12 months prior to enrollment in the study. Approximately half of the participants had been on ART for up to 36 months (n = 215, 50.5%). Nearly half attended ART clinics on a monthly basis (n = 196, 46.0%), and almost all were receiving tenofovir-based ART regimens (n = 414, 97.2%). Viral load (VL) suppression was high, with 407 participants (95.5%) having suppressed VL results. More than half of participants (n = 246, 57.7%) had a CD4 count of less than 350 cells/µL. Most participants reported no comorbid hypertension or diabetes (n = 353, 82.9%). The majority had no kidney disease-related symptoms (n = 409, 96.0%) and no prior diagnosis of kidney disease (n = 416, 97.7%). Information on chronic CKD provided by healthcare providers was perceived as adequate by 393 participants (92.3%). More than half reported having received recommendations for CKD screening during ART follow-up visits (n = 240, 56.3%). In terms of access to services, 264 participants (62.0%) resided within 5 km of a CKD screening facility, and most reported no costs associated with CKD screening (n = 408, 95.8%).

**Table 2:** Clinical characteristics and health information among study participants (N = 426).

Variable	Frequency (n)	Percent (%)
Screened for CKD in the past 12 months		
No	25	5.9
Yes	401	94.1
Time on ART		
Up to 36 months	215	50.5
More than 36 months	211	49.5
Frequency of attending ART clinic		
Monthly	196	46.0
Every 3 months	86	20.2
Every 6 months	144	33.8
ART regimen		
Non-Tenofovir-based	12	2.8
Tenofovir-based	414	97.2
Recent VL results		
Unsuppressed	19	4.5
Suppressed	407	95.5
CD4 Count		
Less than 350	246	57.7
More than 350	180	42.3
Comorbidity (HPT/diabetes)		
No	353	82.9
Yes	73	17.1
Kidney disease-related symptoms		
No	409	96.0
Yes	17	4.0
Prior kidney disease diagnosis		
No	416	97.7
Yes	10	2.3
Perceived adequacy of CKD information from HCPs		
No	33	7.7
Yes	393	92.3
Recommendation of CKD screening at ART follow-up		
No	186	43.7
Yes	240	56.3
Distance from CKD screening facility		
Less than 5KMs	264	62.0
5 to 10 KMs	98	23.0
More than 10 KMs	64	15.0
Costs with CKD Screening		
No	408	95.8
Yes	18	4.2

### Factors associated with CKD screening

Bivariate and multivariate logistic regression analyses of factors associated with CKD screening among the 426 study participants are displayed in Table 3. In bivariate analysis, frequency of ART clinic attendance, CD4 count, and recommendation of CKD screening during ART follow-up were significantly associated with CKD screening. Participants attending ART clinics every six months had significantly lower odds of CKD screening compared with those attending monthly clinics (COR: 0.13, 95% CI: 0.04, 0.39; p < 0.001). Participants with a CD4 count greater than 350 cells/µL had higher odds of CKD screening compared with those with lower CD4 counts (COR; 19.35, 95% CI: 2.59, 144.43; p = 0.004). In addition, participants who reported receiving a recommendation for CKD screening during ART follow-up visits had significantly lower odds of CKD screening compared with those who did not receive such recommendations (COR: 0.10, 95% CI: 0.02, 0.44; p = 0.002).

**Table 3:** Bivariate and multivariate logistic regression analysis of the factors associated with CKD screening (N = 426).

Variable	Category	CKD screening				COR (95% CI)	p-value	AOR (95% CI)	p-value
		No		Yes					
		n	%	n	%				
Age	Less than 40	7	4.2	159	95.8	Ref		–	
	40 and above	18	6.9	242	93.1	0.59 (0.24, 1.45)	0.251	–	–
Sex	Female	18	6.3	266	93.7	Ref		–	
	Male	7	4.9	135	95.1	1.31 (0.53, 3.20)	0.561	–	–
Marital status	No partner	11	6.1	168	93.9	Ref		–	
	Has partner	14	5.7	233	94.3	1.09 (0.48, 2.46)	0.836	–	–
Level of education	Primary/lower	13	6.0	202	94.0	Ref		–	
	Secondary/higher	12	5.7	199	94.3	1.07 (0.48, 0.2.40)	0.875	–	–
Employment	Unemployed	4	5.1	75	94.9	Ref		–	
	Self-/employed	21	6.1	326	93.9	(0.83 (0.28, 2.48)	0.736	–	–
Residency	Peri-Urban	1	1.8	54	98.2	Ref		–	
	Urban	24	6.5	347	93.5	0.27 (0.04, 2.02)	0.201	–	–
Frequency of attending ART clinic	Monthly	4	2.0	192	98.0	Ref		Ref	
	Every 3 months	1	1.2	85	98.8	1.77 (0.20, 16.08)	0.612	1.66 (0.18, 15.41)	0.657
	Every 6 months	20	13.9	124	86.1	0.13 (0.04, 0.39)	<0.001	0.16 (0.05, 0.48)	0.001
Recent VL results	Unsuppressed	1	5.3	18	94.7	Ref		–	
	Suppressed	24	5.9	383	94.1	0.89 (0.11, 6.93)	0.909	–	–
CD4 count	Less than 350	24	9.8	222	90.2	Ref		Ref	
	More than 350	1	0.6	179	99.4	19.35 (2.59, 144.43)	0.004	9.73 (1.21, 78.16)	0.032
Comorbidity (HPT/diabetes)	No	19	5.4	334	94.6	Ref			
	Yes	6	8.2	67	91.8	0.64 (0.25, 1.65)	0.352	–	–
Perceived adequacy of CKD information from HCPs	No	1	3.0	32	97.0	Ref		–	
	Yes	24	6.1	369	93.9	0.48 (0.06, 3.67)	0.480	–	–
Recommendation of CKD screening at ART follow-up	No	2	1.1	184	98.9	Ref		–	
	Yes	23	9.6	217	90.4	0.10 (0.02, 0.44)	0.002	0.17 (0.04, 0.79)	0.024
Distance from CKD screening facility	less than 5KMs	18	6.8	246	93.2	Ref		Ref	
	5 to 10 KMs	6	6.1	92	93.9	1.12 (0.43, 2.91)	0.813	1.33 (0.47, 3.82)	0.592
	More than 10 KMs	1	1.6	63	98.4	4.61 (0.60, 35.19)	0.141	3.72 (0.45, 31.05)	0.226

In multivariate analysis, the three variables remained significantly associated with CKD screening. Attending ART clinics every six months was independently associated with lower odds of CKD screening (AOR: 0.16, 95% CI: 0.05, 0.48; p = 0.001). Higher CD4 count (>350 cells/µL) was independently associated with increased odds of CKD screening (AOR: 9.73, 95% CI: 1.21, 78.16; p = 0.032). Participants who reported receiving a recommendation for CKD screening during ART follow-up visits had significantly lower odds of CKD screening compared with those who did not receive such recommendations (AOR = 0.17, 95% CI: 0.04–0.79; p = 0.024). The Hosmer-Lemeshow goodness-of-fit test yielded a chi-square value of 1.411 with a p-value of 0.994, indicating that the model demonstrated a good fit to the data.

Sociodemographic characteristics, including age, sex, marital status, education level, employment status, and place of residence, as well as viral load status, comorbidity status, perceived adequacy of CKD information, and distance to CKD screening facilities, were not significantly associated with CKD screening in either bivariate or multivariate analyses. Several variables including time on ART, ART regimen used, experience of kidney disease-related symptoms, prior kidney disease diagnosis, and costs associated with CKD screening, were not included in the regression models due to sparse data and zero-cell counts, which resulted in unstable or non-estimable odds ratios.

## 4. Discussion

This study found a high CKD screening prevalence of 94.1% among PLHIV. This finding likely to reflect the routine integration of renal function assessment into HIV care services rather than a formal, guideline-structured CKD screening program. Current HIV kidney care guidelines recommend regular monitoring of serum creatinine and urinalysis among PLHIV receiving ART, particularly during treatment initiation and follow-up [23,25]. Therefore, the observed high prevalence may represent cumulative exposure to routine renal testing among clinic attending patients rather than systematic CKD screening as defined in clinical guidelines. This indicates strong integration of basic renal monitoring within routine HIV care, but suggests variability in how screening is operationalized in routine practice [26]. This uptake is substantially higher than that reported in many low-middle-income (LMI) settings, where CKD screening among PLHIV remains limited due to resource constraints, competing priorities, and inconsistent guideline implementation [27,28]. The observed high coverage may reflect strengthened HIV program infrastructure, increased provider awareness, policy emphasis on routine laboratory monitoring, and effective implementation of differentiated service delivery models that integrate chronic comorbidity screening into HIV care [29]. However, high screening coverage alone is insufficient to ensure optimal outcomes, as the quality of screening, follow-up of abnormal results, and linkage to specialized care remain critical for effective CKD prevention and management among PLHIV [30,31]. In addition to that, this study was conducted in the three urban regional referral hospitals in Dar es Salaam region, Tanzania which are more likely to have better staff, laboratory infrastructure and diagnostic capacity compared with rural and primary health facilities in Tanzania, as evidenced from previous studies conducted in Tanzania shown that staff gap, laboratory service availability and access of diagnostic tests are typically more limited in rural and primary healthcare settings, which could restrict the generalizability of the observed screening prevalence applied to these groups [32,33].

The frequency of ART clinic attendance emerged as a key determinant of CKD screening. Participants attending ART clinics every six months had significantly lower odds of being screened for CKD compared with those attending monthly visits, even after adjustment for potential confounders. This suggests that extended appointment intervals, while beneficial for decongesting clinics and improving convenience for stable patients, may inadvertently reduce opportunities for routine screening of comorbid conditions. Similar observations have been reported in differentiated service delivery models, where reduced clinical contact may limit preventive service uptake if not deliberately integrated into care protocols [34–36]. These findings underscore the need to ensure that CKD screening is systematically incorporated into less frequent ART follow-up schedules.

Also, the current study revealed that a higher CD4 count (>350 cells/µL) was independently associated with increased odds of CKD screening, likely reflecting better health-seeking behavior and stronger engagement with HIV care among immunologically stable individuals. PLHIV with higher CD4 counts generally have well-controlled HIV, consistent ART adherence, and regular clinic attendance, which increases opportunities for comprehensive care, including routine CKD screening [37,38]. Similar findings have been reported in sub-Saharan Africa, where clinically stable PLHIV (CD4 > 500 cells/µL) were more likely to receive preventive services, including renal function testing, due to sustained engagement with care [39]. Provider-related factors may also contribute, as clinicians may prioritize preventive services for patients perceived to be clinically stable and more likely to benefit from early intervention [40]. In contrast, PLHIV with lower CD4 counts may present with competing acute clinical needs, resulting in reduced attention to routine screening. This pattern has been observed in Nigeria, where individuals with advanced immunosuppression (CD4 < 200 cells/µL) were less likely to undergo CKD screening despite elevated risk, as clinical focus shifted toward managing opportunistic infections [31]. These findings underscore the importance of integrating NCD screening into HIV care across all levels of immunological recovery and highlight the need for targeted strategies to ensure that immunocompromised patients are not overlooked in routine CKD screening.

There was an inverse association between healthcare provider recommendations for CKD screening and actual screening uptake among PLHIV, which was discovered in the present study. Although this finding appears counterintuitive, it may reflect reverse causality [41,42], in which healthcare providers are likely to recommend screening to patients perceived to be at higher risk or with advanced disease, groups that may already have lower screening uptake due to competing clinical priorities [31]. Furthermore, residual confounding, namely disease severity, comorbid conditions, or access-related barriers that impact both the likelihood of receiving a healthcare provider recommendation and actual screening uptake, may influence this association [42,43]. Additionally, contextual barriers within the study setting may have reduced the effectiveness of provider recommendations. The clinics in which this study was conducted had a high patient-to-staff ratio, limited private space for counselling, and insufficient time for individualized health education and advice [21]. These structural restrictions may hinder effective provider-to-patient communication, patient understanding, and timely access to screening services, thereby contributing to lower screening uptake regardless of the recommendations. Related studies from HIV clinics in sub-Saharan Africa have shown that prolonged waiting times, staffing shortages, and privacy-related challenges adversely affect service delivery and uptake of recommended care services [44–48]. Therefore, this finding, which underscores that provider recommendation alone is insufficient and highlights the need for integrated, system-level approaches to ensure effective CKD screening within routine HIV care, should be considered hypothesis-generating and warrant further investigation in longitudinal studies.

The sociodemographic characteristics were not significantly associated with the uptake of CKD screening. Similarly, viral load status, comorbidity with hypertension or diabetes, perceived adequacy of CKD information, and distance to CKD screening facilities showed no significant associations. These findings suggest relatively equitable access to CKD screening within the study population and may reflect the strengths of Tanzania’s ART program in minimizing social and structural barriers to care. Comparable findings were reported in Dar es Salaam, where age, sex, education level, and comorbidity were not significantly associated with screening for NCDs among PLHIV on ART [49]. However, evidence from other settings differs; studies in Uganda reported higher education levels to be associated with missed NCD screening opportunities [50], while research in Kilimanjaro demonstrated a negative association between distance to health facilities and cervical cancer screening, another NCD-related service [51]. These variations highlight the context-specific nature of NCD screening behaviors and underscore the influence of health system organization and service integration on CKD screening uptake.

Several limitations should be considered when interpreting the findings of this study. First, the cross-sectional design precludes causal inference, and observed associations should be interpreted as correlational. Second, reliance on routine clinic records and self-reported information may have introduced information bias, including misclassification or recall bias. In addition, CKD screening was operationally defined as documentation of at least one renal assessment test since ART initiation, which may reflect cumulative routine renal monitoring rather than a formal, guideline-structured CKD screening program, potentially contributing to the high observed screening prevalence. Third, the presence of sparse data and zero-cell counts for several clinically relevant variables limited their inclusion in regression analyses, potentially underestimating their true association with CKD screening. Finally, the study was conducted within ART clinics in the urban referral hospitals, which may limit the generalizability of findings to populations with limited engagement in HIV care or to non-HIV settings, and to rural and primary healthcare facilities, where staffing, diagnostic capacity, and access to routine renal monitoring may be more constrained.

## 5. Conclusion

CKD screening uptake among adults receiving ART was high; however, important differences were observed based on frequency of ART clinic attendance, CD4 count, and provider recommendation. Patients with less frequent ART visits were significantly less likely to undergo CKD screening, highlighting a potential gap in preventive service delivery within differentiated ART models. Strengthening provider-initiated screening recommendations and integrating CKD screening into all ART follow-up schedules, regardless of visit frequency or immunological status, may enhance early detection and management of kidney disease among people living with HIV. These findings support the need for deliberate, effective integration of NCD screening into HIV care to sustain long-term health outcomes.

## Supporting information

S1 Dataset Study dataset of CKD screening among PLHIV. (XLSX)
